# Monolithic integration of one VCSEL on a single mode fiber

**DOI:** 10.1515/nanoph-2025-0047

**Published:** 2025-06-23

**Authors:** Federica Piccirillo, Michael Zimmer, Martino Giaquinto, Alberto Micco, Michael Jetter, Peter Michler, Andrea Cusano, Simone Luca Portalupi, Armando Ricciardi

**Affiliations:** Optoelectronics Group, Department of Engineeing, 18952 University of Sannio, Benevento, Italy; Institut für Halbleiteroptik und Funktionelle Grenzflächen, Center for Integrated Quantum Science and Technology (IQST) and SCoPE, University of Stuttgart, Stuttgart, Germany; Department of Information Engineering and Electrical Engineering and Applied Mathematics, University of Salerno, Fisciano, Italy

**Keywords:** optical fibers, laser, VCSEL, active fibers, lab-on-fiber technology, optoelectronics on fiber technology

## Abstract

The implementation of compact fiber-coupled light sources and devices represents a highly sought through technological goal, in wearable technologies, point-of-care units, telecommunication, and even quantum technology. In particular, a strong reduction of the overall device footprint, still ensuring a compact electrical contacting, would play an important role for electrically driven and electrically controlled devices. Here we show the integration of electrically pumped vertical-cavity surface-emitting lasers on multi-mode and single-mode fibers. The optimized integration technique is enabled by the advanced fiber-to-laser coupling design allowed by a detailed numerical investigation, as well as by an improved technological approach. While for the integration on multimode fibers, an important improvement over state-of-the-art is achieved, the integration on single-mode fiber is here demonstrated for the first time. All experimental results include reproducibility studies to show that the developed technique can be considered for larger scale implementations and are further supported by numerical investigation. This work marks an important step forward in the miniaturization of fiber-based optoelectronics devices which will be highly beneficial for various research and technology developments.

## 1 Introduction

Historically, optical fibers have assumed a ‘passive’ role in the first decades after their discovery, being generally associated with a means of transmitting optical signals from a source to a detector at high bit rates and low attenuation [1]. Over time, the scientific community has slowly transformed fiber optics, giving them a more ‘active’ role, by making them useful not only for transport but also for information detection [2], [3]. Fiber-optic sensing is now a well-established technology with strong spin-offs in the industry [4]. An important example is the lab-on-fiber technology that has revolutionized the world of fiber optic sensing by transforming the tip, the side surface, or the inner volume of the fiber into a miniaturized laboratory [5], [6], [7]. Micro- and nano-manufacturing techniques have been customized to become a useful tool for integrating on the common optical fiber components and materials for the purpose of giving them the characteristics of multifunctionality [8].

Regardless of the field of application and the complexity of fiber optic devices, they make use of external sources to excite in fiber functional materials, and external detectors to monitor propagated light [9]. Standard optical fiber tools and equipment can be bulky and require additional on-chip optics, resulting in complex, non-portable systems. For this reason, in the last years, the scientific community has shown a growing interest in developing all-in-fiber compact optoelectronic platforms [10]. According to this increasing interest, the “Optoelectronics on Fiber Technology” could be set as a novel vision featuring advanced fiber-based integrated circuits combining the unique characteristics of optical fiber (small size, lightweight, flexibility, high transmission efficiency, biocompatibility) with miniaturized advanced optoelectronic materials and components, exploiting the intrinsic light (in- or out-) coupling property of the fiber endface [11]. Actively controllable multifunctional all-fiber devices combining emission, detection, modulation, and sensing capabilities in a single unit with a compact footprint, could represent a technological and scientific breakthrough in many application fields. As an example, the possibility to squeeze the entire optical interrogation setup (source, fiber, detector) into a single compact and monolithic unit can promote the adoption of wearable and assistive technologies which, although well developed from the monitoring device point of view, are lacking in interrogation systems that are still bulky and heavy, and therefore impractical [12].

The first step towards obtaining this new generation of all-in-fiber miniaturized optoelectronic platforms is developing a reliable procedure allowing to integrate and contact optoelectronic materials and/or chips to the fiber with high efficiencies and within a CMOS compatibile process.

So far the proposed techniques have followed two different main paths [11]. One category is the “multimaterials fibers” technology in which multifunctional fibers are created by means of a thermal drawing process from multimaterial preforms on a micrometer scale [13]. Multimaterial fibers nowadays find various applications in many contexts including optogenetics [14]. The second approach essentially concerns the integration of 2D optoelectronic materials (in most cases graphene-based films or similar) onto conventional or specialty silica fibers [15]. Similarly to what happened with the lab-on-fiber technology, this second approach has made it possible to divide the “optoelectronics on fiber technology” into three subcategories: i) optoelectronics around fiber (when the optoelectronic material is integrated on the lateral surface of microfibers [16], D-shaped fibers [17], or reduced core curved fibers [18] enabling evanescent field interaction), ii) optoelectronics in fiber (when the electro-optical material is integrated inside the holes of photonic crystal fibers [19], [20] or inside a standard fiber [21] and iii) optoelectronics on tip (when the termination of the fiber becomes the substrate on which to place the optoelectronic component) [15], [22]. Interesting examples of optoelectronics on fiber technology have recently also concerned the use of multicore fibers to obtain the possibility of multiplexing [23]. However, most of the approaches proposed so far allow for the integration of photodetectors or modulators but not optical sources (LED or LASER) monolithically integrated into the fiber.

In this framework, we have recently proposed an optoelectronics on tip approach based on the combination of both direct and indirect fabrication of optoelectronic chips onto the optical fiber tip [24], [25]. The approach involves the use of the lab-on-fiber technology for the preparation of the electrode structure achieved thanks to a gold layer directly deposited onto the fiber facet. Successively, UV laser micromachining is applied for making apertures in correspondence of the fiber core. At the same time, the optoelectronic chip is fabricated with conventional photolithographic and etching steps from a planar substrate and then transferred, aligned, and bonded to the fiber end-face. By following this approach, we reported a proof-of-concept active fiber device resulting from the monolithic integration of a compact electrically driven quantum-well vertical-cavity surface-emitting lasers (QW-VCSELs) on a multi-mode fiber (MMF) [24]. The overall in-coupling efficiency on MMF was limited to 20 %, due to several technological non-idealities of the fabrication protocol (in particular the fiber-to-laser alignment) which ruled out the possibility of coupling VCSEL to a single-mode fiber (SMF), which is a desirable feature in many applications [26].

In this work, we propose and experimentally verify a new optimized semi-automized process to integrate any compact optoelectronic chip on the fiber end-face, easily extendible to mass production thanks to its compatibility with standard industry processes. The design rules of the new process have been here derived from the results of a numerical model that allows us to calculate the theoretical coupling efficiency under different integration conditions. The numerical method enabled us to understand the limiting aspects of the previous approach and provided new insight on how to overcome them with a newly optimized integration procedure. Specifically, we used a single miniaturized VCSEL, thus simplifying the alignment procedure with the fiber’s core and enabling direct control of the injected current. Moreover, the VCSELs were bonded on the fibers through thermo-compression, by using a die-bonder (Fineplacer^®^ lambda 2 [27]), minimizing the VCSEL-to-fiber distance and the coupling losses. The technological optimization of the integration procedure allows to achieve a VCSEL-to-MMF coupling efficiency higher than 90 %, improving the performance of the device by more than a factor of four with respect to previous state-of-the-art. Moreover, with the new integration procedure, we were also able to successfully integrate for the first time a VCSEL onto a SMF, measuring an in-coupling efficiency of more than 40 %. Translating our integration procedure on SMF represents a breakthrough for fiber-integrated devices, paving the way for direct integration of the electrically and/or optically driven sources and detectors on the fiber end-face with high throughput, high mechanical stability, high coupling/detection efficiency, monolithic electrical contact, and an unprecedented degree of miniaturization.

## 2 Results

### 2.1 Coupling efficiency: numerical modeling

A numerical method for calculating the coupling efficiency between the laser and the fiber was developed with the dual purpose of understanding the limitations of our previous approach and getting guidance on how to improve the entire integration procedure. The method takes as input both the optical fiber parameters (core radius, numerical aperture) and laser features (beam waist, divergence angle, mode profile) and calculates the overlap integral between the modes allowed in the fiber and the laser beam distribution. Specifically, the coupling efficiency $\eta \left(E_{{\left(n,m\right)}_{L}\text{,}}E_{{\left(l,p\right)}_{F}}\right)$ between a laser $E_{{\left(n,m\right)}_{L},}$ , and fiber $E_{{\left(l,p\right)}_{F}}$ mode electric field is defined as [28]:

(1) $$\begin{array}{ll} & \eta \left(E_{{\left(n,m\right)}_{L}},E_{{\left(l,p\right)}_{F}}\right)\\ & \;=\frac{{\left|\iint E_{{\left(n,m\right)}_{L}}\left(x,y\right){E^{*}}_{{\left(l,p\right)}_{F}}\left(x,y\right)\text{d}x\text{d}y\right|}^{2}}{{\iint \left|E_{{\left(n,m\right)}_{L}}\left(x,y\right)\right|}^{2}\text{d}x\text{d}y{\iint \left|E_{{\left(l,p\right)}_{F}}\left(x,y\right)\right|}^{2}\text{d}x\text{d}y} \end{array}$$

where the subscripts couples n,m and l,p indicate the laser TEM mode number and the fiber linearly polarized propagation modes, respectively.

In the case of SMF only the fundamental mode (n = m = 0) is supported, that is a Gaussian beam. In the case of step-index MMF, the analytical solutions of the supported lp modes can be expressed as combinations of Bessel functions (and modified Bessel functions) in the fiber core (and the cladding). The number of supported modes is related to both its geometrical (core diameter) and physical (refractive index contrast) properties (see the supporting information (SI) for more details). Consequently, the total modal coupling efficiency of a specific VCSEL mode $E_{{\left(n,m\right)}_{L}}$ to the MMF can be calculated [28] by summing all the single coupling efficiencies of all the guided fiber modes (lp), i.e.

(2) $$\eta_{\text{MMF}}=\overset{l_{\max}}{\underset{l=0}{\sum }}\overset{p_{\max}}{\underset{p=1}{\sum }}\eta \left(E_{{\left(n,m\right)}_{L},}E_{{\left(l,p\right)}_{F}}\right)$$

**Figure 1:**
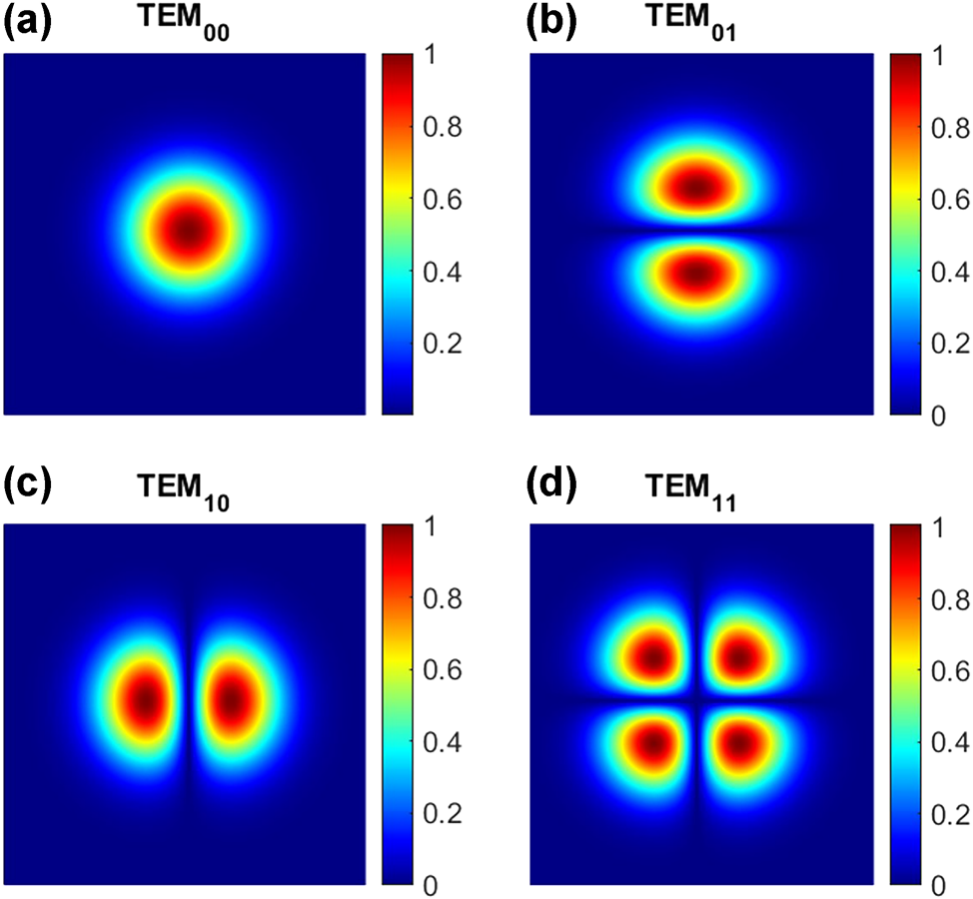
Simulations of the first four normalized Hermite-Gaussian modes of a VCSEL.

VCSEL transverse higher-order modes can be described using either Laguerre–Gaussian (LG) mode functions or Hermite–Gaussian (HG) ones, whenever the laser cavity shows a cylindrical or a rectangular symmetry, respectively. For our numerical calculations, we preferred to model the VCSEL higher-order modes as HG TEM_*nm*_ solutions, which can be written in the form [29]:

(3) $$E_{{\left(n,m\right)}_{L}}\left(x,y,z\right)=E_{{\left(n\right)}_{L}}\left(x,z\right)\times E_{{\left(m\right)}_{L}}\left(y,z\right)$$

$$\begin{array}{ll} E_{{\left(n\right)}_{L}}\left(x,z\right) & ={\left(\frac{2}{\pi }\right)}^{\frac{1}{4}}{\left(\frac{\exp\left[j\left(2n+1\right)\psi \left(z\right)\right]}{2^{n}\text{n}!\omega \left(z\right)}\right)}^{\frac{1}{2}}\\ & \;\times H_{n}\left(\frac{\sqrt{2}x}{\omega \left(z\right)}\right)\exp\left[-jkz-j\frac{kx^{2}}{2R\left(z\right)}-\frac{x^{2}}{\omega^{2}\left(z\right)}\right] \end{array}$$

$$\begin{array}{ll} E_{{\left(m\right)}_{L}}\left(x,z\right) & ={\left(\frac{2}{\pi }\right)}^{\frac{1}{4}}{\left(\frac{\exp\left[j\left(2m+1\right)\psi \left(z\right)\right]}{2^{m}\text{m}!\omega \left(z\right)}\right)}^{\frac{1}{2}}\\ & \;\times H_{m}\left(\frac{\sqrt{2}y}{\omega \left(z\right)}\right)\exp\left[-jkz-j\frac{ky^{2}}{2R\left(z\right)}-\frac{y^{2}}{\omega^{2}\left(z\right)}\right] \end{array}$$

where *ω*(*z*) is the spot size, *R*(*z*) is the wavefront radius of curvature, $\psi \left(z\right)=tan^{-1}\left(z/z_{r}\right)$ is the Guoy phase shift, and *H*_*n*,*m*_ are the Hermite polynomials of order n,m. Normalized beam profiles of the TEM_00_, TEM_01_/TEM_10_ and TEM_11_ are shown in Figure 1.

Increasing the forward current of the VCSEL increases the number of excited transverse modes. The laser mode field, at a specific current value, is given by a linear combination of different HG modes [30]. Moreover, higher values of injected current lead to an increase of the VCSEL beam divergence angle θ and as a consequence the reduction of the beam waist.

According to these considerations, for both SMF and MMF, we carried out a numerical study (Matlab, R2023a) aimed at evaluating the dependence of the coupling efficiency variation on (i) the laser beam waist ω (in the range 0.5–10 μm at a distance of z = 0 µm) and (ii) the laser-to-fiber gap z (in the range 0–30 µm for a waist of 2.5 µm). These values were chosen keeping in mind the real application context of this study. For each combination of distance and waist, we calculated the coupling efficiency by solving the overlap integral described in Equation (1). We performed the calculations for the fundamental mode $\left(\text{TE}{\text{M}}_{00}\right)$ and also for three higher-order modes $\left(\text{TE}{\text{M}}_{01},\text{TE}{\text{M}}_{10},\text{TE}{\text{M}}_{11}\right)$ .

The results of numerical analysis indicate that the coupling efficiency shows different trends for SMF and MMF. In the case of MMF, the data indicate that a specific threshold value for the waist to reach the maximum coupling efficiency; below that value the divergence angle is so large that it does not favor beam coupling within the fiber. Obviously, different modes exhibit similar trends due to the intrinsic characteristics of MMF. In the case of SMF, the trend of coupling efficiency as a function of the waist is more complex; the trend presents a maximum at a specific waist value that depends on the laser mode. As expected, the efficiency is highest for the TEM_00_ mode case with the maximum being obtained for a waist of about 2.5 µm, which matches the mode field radius (MFR) of the fiber.

**Figure 2:**
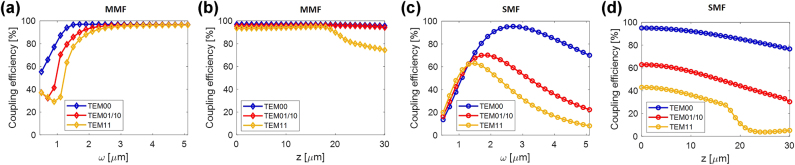
Parametric studies of the theoretical coupling efficiency for MMF (above) and SMF (below) as a function of the waist (a, c), and distance (b, d). TEM_01_ and TEM_10_ are consistently overlapping as expected.

Figure 2 shows the coupling efficiency trends as a function of the distance between the laser and the fiber, for a fixed waist value. For both fibers (SMF and MMF) the trend is monotonically decreasing, signifying that it is preferable to reduce the gap between laser and fiber as much as possible to maximize coupling (still being in far-field). Moreover, different modes exhibit different trends and the differences in coupling efficiencies with respect to the fundamental one amplify as the gap increases. Specifically, the decreasing behavior for MMF has a more pronounced slope for higher-order modes compared to the TEM_00_ mode, especially TEM_11_ with a reduction of about 30 % for distances bigger than 20 µm. This is even more evident for the SMF with a difference of more than 50 % between the fundamental mode and the TEM_11_ for small distances (z < 5 µm), which increases to about 80 % at z = 30 µm. It is important to emphasize that the trends shown in Figure 2 strongly depend, even quantitatively, on the combination of waist and gap and also on the characteristics of the fiber and laser used.

The results of the numerical analysis suggest two important design rules to be taken into account for the optimized fabrication procedure. First, it is desirable to maintain at minimum (potentially zero) the distance between the laser and the fiber. In that case, the coupling efficiency is maximized and the dependence on modal dispersion of the laser beams due to increased injection current is minimized. Concerning the waist, it is necessary to limit the divergence angle for MMF and choose a beam waist with a Gaussian profile that matches the MFR of the SMF. The MFR can be controlled by acting on the size of the VCSEL oxide apertures. Therefore, these considerations essentially suggested to make the following modifications with reference to the previous integration process demonstrated in Ref. [24]: (i) to develop a new bonding technique overcoming the use of conductive epoxy glue (that creates a 30 µm gap between the chip and the fiber), and (ii) to optimize the chip layout, keeping the nominal oxide aperture on the smaller side for achieving a Gaussian beam and maximize coupling efficiencies for both SMF and MMF.

### 2.2 The optimized integration method

In the wake of the results of the parametric analysis, we developed an optimized integration procedure consisting of three main steps: the fiber preparation, the chip fabrication, and the bonding of the chip onto the fiber tip. Each step was tailored in order to enhance the VCSEL-to-fiber coupling efficiency, thus making the integration procedure more reliable and effective. Below we provide a brief discussion of each of these fabrication phases, while more technical details are reported in the Experimental Section.

#### 2.2.1 The fiber tip preparation

Both MMF (core diameter of 105 µm and a numerical aperture (NA) of 0.22) and SMF (core diameter of 4 µm and a NA of 0.1, fiber for the visible spectral range) with step-index profile were used for the experiments. Regarding the optical fiber preparation, a 10 nm thick titanium adhesion layer, followed by a gold layer with a thickness of 150 nm, were deposited on both the facet and the lateral surface of the fiber through electron beam evaporation. A UV laser micro-machining process was then employed to open a circular aperture in correspondence to the fiber core. No alignment markers were defined since the small dimension of the core of the SMF in the visible range (4 µm) caused more restrictive margins for the alignment of the VCSEL (see the Section 2.2.3 on the chip-to-fiber integration procedure). Finally, a galvanization process was exploited to increase the thickness of the gold layer in preparation for the chip (thermo-compression) bonding. The thickness of the final gold layer was estimated to be around 2 µm.

#### 2.2.2 The chip fabrication and characterization

**Figure 3:**
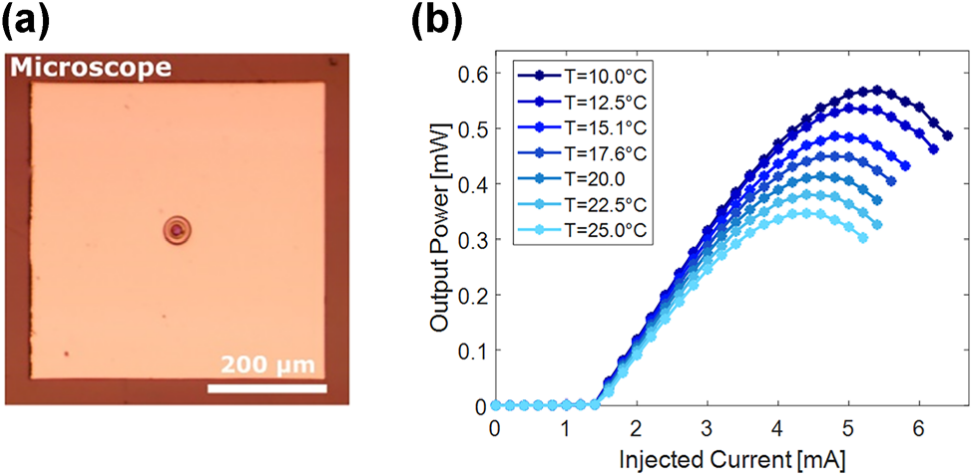
Characteristics of a selected VCSEL. (a) Microscope image (top view) of a cleaved sample. (b) Power characterization of a VCSEL in free space for different heatsink temperatures.

For this study, we have selected an electrically driven QW-VCSEL emitting in the red spectral range, with an emission wavelength of 670 nm. Worth noting, the new design involves the use of only one emitting VCSEL (shown in Figure 3a), instead of four VCSELs as for the proof-of-concept device [24], thus allowing the control of the injected current on each laser device. The chip was fabricated in a standard process for oxide-confined VCSELs (see Methods and Materials) and then cleaved into pieces of about 500 µm × 500 µm (shown in Figure 3a), to minimize their footprint compared to the diameter of the fiber ferrule (2.5 mm). According to our numerical study previously discussed, a nominal oxide aperture diameter of 4 µm was chosen as a good compromise between Gaussian mode emission and maximum achievable emitting power [31].

After fabrication (and before integration on the fiber), each VCSEL was placed on a thermal heat sink and contacted with electrical probes to measure their emitted power as a function of the injected current (shown in Figure 3b and S4 of the SI), at different temperatures. The VCSEL emission spectra (shown in Figure S3 of the SI) were measured for different heat sink temperatures to determine the working temperature of the device after the integration on the fiber [24]. Finally, the beam profiles of the VCSELs were measured at two specific values of the injected current, in particular after the VCSELs started lasing and near the thermal rollover.

**Figure 4:**
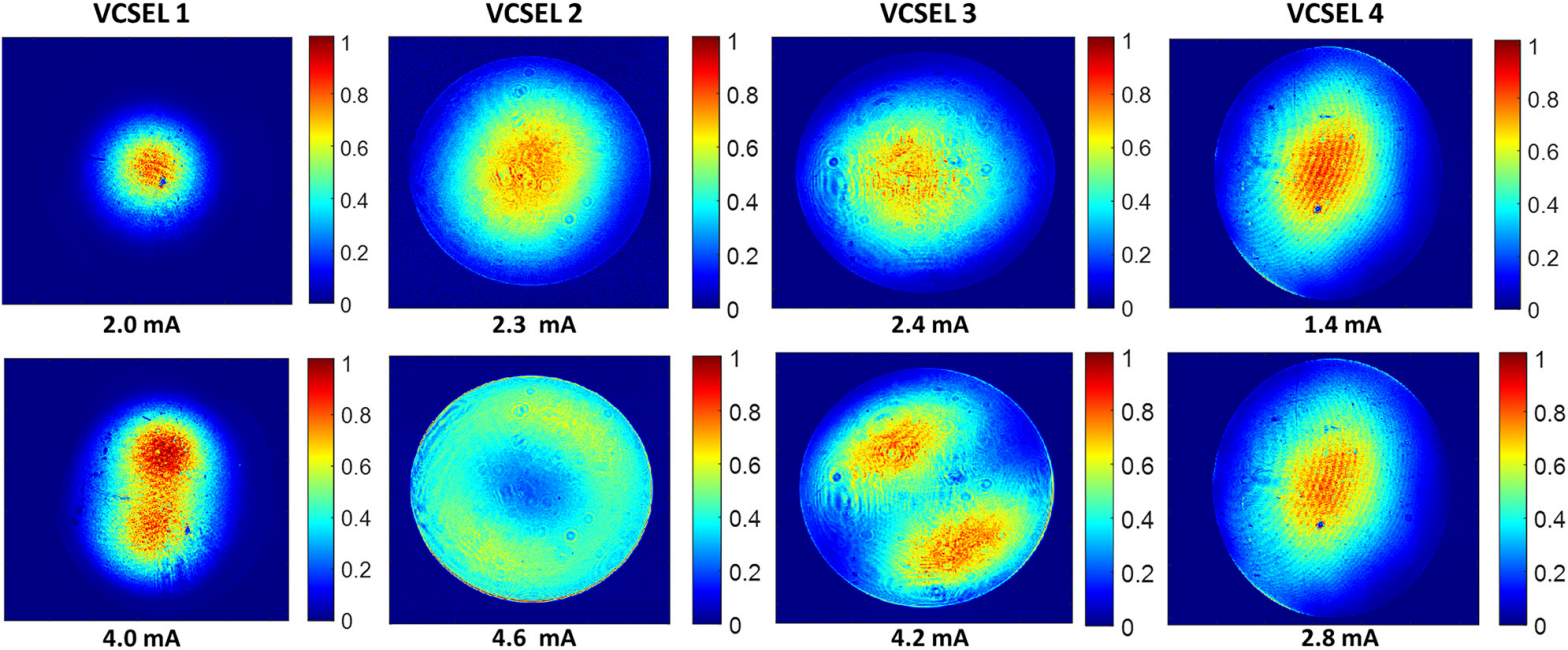
Beam profile of the four VCSELs selected for the integration, at two different current values and at a temperature of 15 °C.

In our experiments, we have selected four VCSELs, whose beam profiles are shown in Figure 4. The oxide apertures diameters of the fabricated VCSELs are estimated to be around 5.5 µm for VCSEL 1 and 2, and 3 µm for VCSEL 3 and 4, with a slight ellipticity. All fabricated VCSELs exhibit a Gaussian mode for low values of injection current. As expected, most of the devices show a variation of the beam profile for increasing currents. Specifically, VCSELs 1 and 4 maintain a quasi-Gaussian distribution even at roll-over, while VCSEL 2 and 3 exhibit a two peaks toroidal-like shape, given by the combination of TEM_01_ and TEM_10_ [29]. According to these characteristics, in order to validate experimentally the observations resulting from the numerical analysis (having two different types of beam profiles integrated on each fiber), we determined to integrate the first two (1 and 2) VCSELs on the MMF and the last two (3 and 4) on the SMF, so that both profile types are tested on the two fibers.

#### 2.2.3 The chip-to-fiber integration procedure

**Figure 5:**
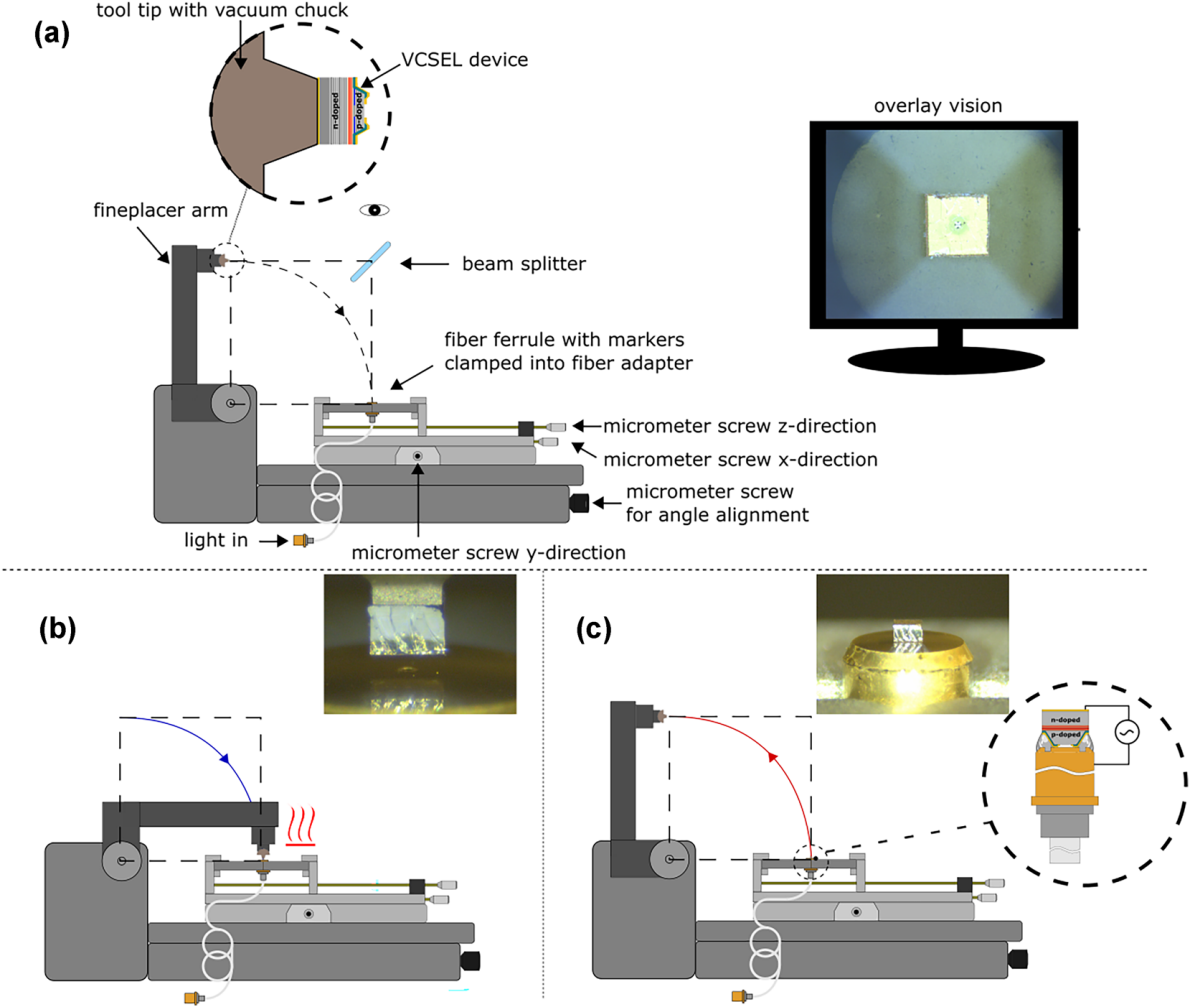
Illustration of the new bonding procedure with the bonder Fineplacer^®^ lambda 2. (a) The VCSEL chip is picked up up-side-down via a vacuum chuck at the tool tip of the fine-placer arm. The fiber onto which the integration is intended is clamped into a customized adapter on the fineplacer table. By using micrometer screws the fiber can be aligned with regard to the picked up VCSEL sample by observation over a monitor via the overlay vision alignment system. (b) The fineplacer arm is put down, bringing the VCSEL chip into contact with the fiber ferrule and the thermo-compression bonding procedure is initiated. (c) After the thermo-compression bonding process, the vacuum of the tool tip is turned off and the fineplacer arm raised, leaving behind the VCSEL chip bonded to the fiber ferrule.

A new semi-automated procedure was used to integrate the VCSEL on the termination of the optical fiber. The entire process is schematically shown in Figure 5. Instead of manually transferring and bonding the chip onto the fiber, here we optimized the integration step by employing the table top flip chip bonder Fineplacer^®^ lambda 2 equipped with an overlay vision alignment system (VAS) [27]. To improve the chip-to-fiber alignment precision for the SMF, we illuminated the fiber via the other termination to correctly identify its core on the VAS before the bonding step. Then, the VCSEL is accurately approached to the fiber up to contact and thermo-compression ensures the bonding. The thermo-compression process ensured the electrical contact between the p-contact of the VCSEL and the gold coating deposited on the fiber ferrule. This procedure allowed us to avoid the use of conductive epoxy glue on the fiber ferrule, avoiding ohmic losses and bubble formation that may have otherwise occurred.

The reliability of the process was demonstrated by the successful integration of two VCSELs (VCSEL 1 and 2) on MMFs and two other VCSELs (VCSEL 3 and 4) on SMFs, respectively shown in Figure 4.

**Figure 6:**
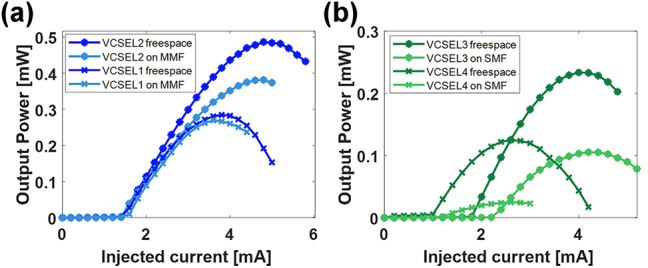
Power as a function of current measured in free space (darker curves) and after the integration (light blue and green curves) on (a) MMFs and (b) SMFs, respectively. Symbols correspond to the same laser device before and after integration.

After the VCSEL integration on the fiber, the device was contacted with the customized electrical PCB (with the copper block contacting the gold coated fiber ferrule and a needle to contact the backside of the VCSEL, as shown in Figure S2 of the SI) suitably realized to control the current injection into the laser. Analogously to the free space characterization, we measured the emitted power as a function of the current (see method section). Figure 6a and b shows the output powers as a function of the injected currents collected out of the MMF and SMF, respectively. As a reference, we also show the power levels emitted by the VCSEL before the fiber integration (i.e. in free space).

**Figure 7:**
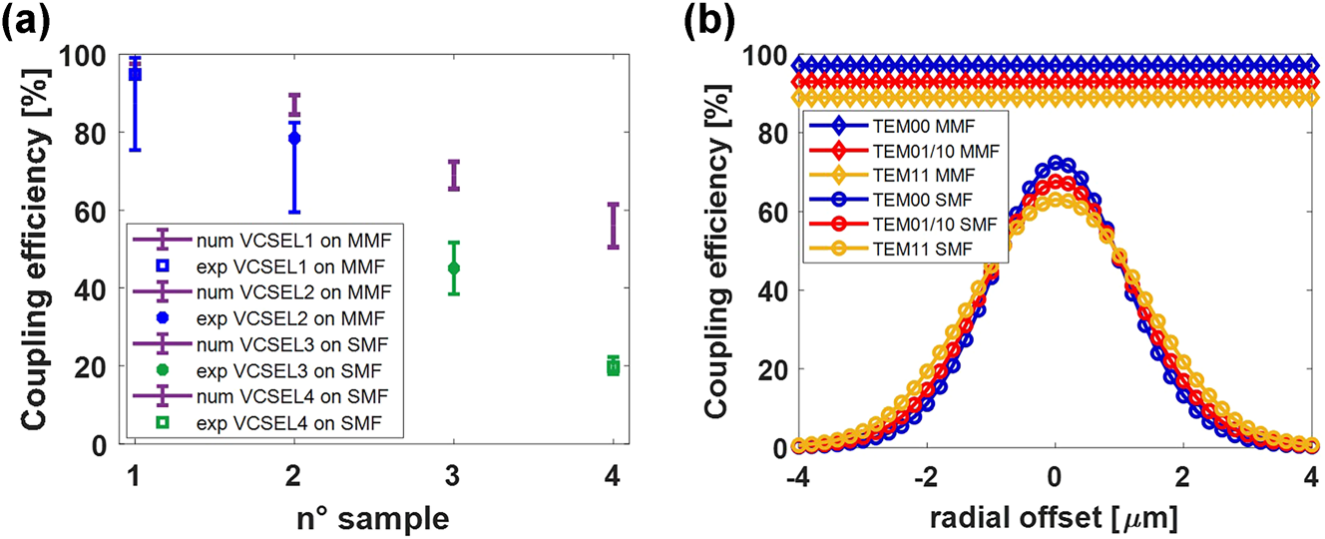
Coupling efficiencies. (a) Comparison between theoretical and experimental coupling efficiencies for all devices at thermal rollover. (b) Numerical study of the theoretical coupling efficiency for MMF (diamond markers) and SMF (circle markers) as a function of radial offset, (TEM_01_ and TEM_10_ are consistently overlapping as expected).

For the MMF, a maximum coupling efficiency at thermal rollover in the order of 95 % was achieved, improving the performance with respect to our previous work of more than a factor of 4. Repeatability analysis on other MMFs confirmed coupling efficiencies within the 80 % range. Furthermore, we successfully achieved for the first time the VCSEL-to-SMF monolithic integration with a maximum coupling efficiency of about 45 %. Repeated integration procedures of other VCSEL on SMFs resulted in coupling efficiencies within the 20 % range. Laser integrations on SMF require higher alignment precision due to the small size of the core radius which is similar to the nominal size of the VCSEL oxide apertures (more details are provided in the following section). Overall the fiber integrated devices have not shown degradation during the characterization measurements and the essential features of the VCSELs are not affected by the procedure. However, the slight variation of both the threshold and rollover currents (before and after the integration) can be attributed to slight changes in the structural characteristics of the laser as a result of the thermo-compression process. This is particularly visible in the case of VCSEL 4 which is, not surprisingly, characterized by the lowest efficiency (shown in Figure 7).

## 3 Discussion

In Table 1 we evaluated the experimental coupling efficiency by considering the power levels at the thermal rollover; this allows a fair analysis that takes into account all the variations in the characteristics of the VCSEL before and after the chip-to-fiber bonding. Since the temperature at which the laser operates when it is integrated on the fiber termination is not easily quantifiable, the coupling efficiency has been also evaluated for different temperature values in the range of 10–15 °C. The results are shown in Figure 7a for both the two MMFs (in blue) and the two SMFs (in green), where the maximum/minimum coupling efficiency values correspond to the maximum (15 °C)/minimum (10 °C) temperature. To make a comparison with the theoretical data, in the same figure we also calculated the values of the theoretical coupling efficiency by exploiting the model discussed in Section 2.1. To this aim, for each VCSEL, we derived from experimental observations the beam waist, the modal distribution, and the gap between the chip and the fiber. The beam waist was estimated for each VCSEL, by taking into account the experimental beam profiles and the oxide aperture diameter [32] (see the SI). In particular, we estimated a waist of 1.9 µm, 1.5 µm, 1.6 µm and 1.2 µm for VCSEL 1, 2, 3, and 4 respectively, considering a variation of ± 0.1 µm and showing the results as error bars in the graph. Laser beam profiles were also fitted with different combinations of percentages of TEM_00_, TEM_01_, and TEM_10_ modes (shown in Figure S8 of the SI). For the sake of clarity, since the experimental observations do not allow us to derive the precise value of these percentages, they were varied to the extent of 10 percent (shown as error bars in Figure S9 of the SI) showing a limited influence on the coupling efficiency. Finally, we selected a chip-to-fiber gap of 3 µm for all four devices, taking into account the thickness of the gold layer after the galvanization procedure. In any case, variations in the order of a few microns of that gap do not affect too much the coupling efficiency (shown in Figure 2b and d).

**Table 1:** Power measured at thermal rollover with respective current, in free space and after the integration on the fibers, and coupling efficiency.

VCSEL n°	Free space @ rollover		On-fiber @ rollover		η
	P (µW)	I (mA)	P (µW)	I (mA)	
MMF 1	284	3.8	269	3.6	94.7 %
MMF 2	486	4.8	381	4.8	78.4 %
SMF 3	233	4.0	105	4.2	45.0 %
SMF 4	125	2.6	25	2.6	19.7 %

The results of our calculations are shown in Figure 7a (magenta points). For MMF the numerical-experimental agreement is very good given the greater robustness of the coupling efficiency with respect to the variation of laser parameters. The coupling efficiency of VCSEL 1 is higher than that of VCSEL 2 because it has a more Gaussian modal distribution (shown in Figure 2a). In the case of SMF, although VCSEL 4 has a more Gaussian profile, the lower coupling efficiency relative to VCSEL 3 is due to its smaller waist size. In fact, with reference to Figure 2c, a decrease in the waist from 1.5 µm to 1 µm causes a 30 % reduction in coupling efficiency. The residual disagreement between numerical and experimental results in the case of SMF is attributable to the misalignment of the VCSEL with respect to the fiber core during integration. In fact, the SMF is by its nature much more sensitive to misalignment, having a smaller core and sustaining only one mode upon propagation. In particular, the SMF used has a radius of only 2 µm, which makes the alignment process particularly challenging.

To quantify the impact of lateral misalignment on the integration procedure, the numerical model was used to calculate the percentage decrease in coupling efficiency in case of radial displacement in the x, y plane between the laser and the fiber in the range 1–4 µm. Figure 7b shows the calculated coupling efficiencies as a function of the radial offset obtained for the different modes in the case of MMF and SMF, respectively. As expected, thanks to its large core radius (about 25 times larger than the VCSEL oxide aperture) the MMF coupling efficiency exhibits a constant trend. Different case for the SMF for which a radial offset of only 1 µm is enough to cause a reduction in coupling efficiency of about 50 %. The data in Figure 7b also show that higher-order modes are more robust than the fundamental mode with respect to misalignments between laser and fiber. These results show why the experimental-numerical disagreement of VCSEL4 is greater than that of VCSEL3.

The experimental coupling efficiency can also be estimated for each current value (shown in Figure S5 of the SI). However, the coupling efficiency value calculated in this way is affected by changes in the threshold current resulting from integration, especially for low powers (or currents). To circumvent this, the coupling efficiency was estimated for each value of emitted power normalized with respect to that relative to rollover (shown in Figure S6 of the SI).

Overall, these results show that the optimized process led to a significant increase in coupling efficiency and also enabled monolithic integration of chips on SMF. In the latter case, the coupling efficiency strongly depends on laser beam profile characteristics that need to be carefully controlled. Moreover, the repeatability of the process is influenced by the sensitivity of the coupling efficiency with respect to the radial misalignment. This can be corrected by improving the accuracy of the automated alignment process (the current limitation is on the magnification of the VAS) and by integrating a lens such as a metasurface on the tip of the SMF enhancing the coupling efficiency even when a radial offset is present [33].

## 4 Conclusions

In this work, we demonstrated a new optimized semi-automated fabrication process that allows optoelectronic chips to be monolithically integrated onto the end face of the optical fiber. Our procedure is flexible since it can be applied to different types of miniaturized components (such as optical sources and detectors) and of fibers, including speciality fibers, since the fiber preparation and integration steps remain the same. In the specific case study, the method has been applied to integrate VCSEL emitting at 670 nm on both MMF and SMFs. Compared with our previous work, we introduced significant improvements that enabled us to bring the coupling efficiency up to ∼90 % in the case of MMF. Moreover, we have also demonstrated for the first time the coupling of a VCSEL within single-mode fiber, achieving coupling efficiencies up to 45 %. The coupling efficiency, especially in the case of SMF, can be improved by acting on several aspects concerning (i) further optimization of the alignment process, (ii) improvement of the Gaussian shape of the laser beam, and (iii) increase of the numerical aperture of the collecting fiber. Regarding the first point, it is important to note that the resolution of the integration step (set by the visualization optics) turns out to be comparable with the radius of the fiber core. A customization of the die bonder with improved performance (enhanced optical magnification) could achieve better alignment and bonding, and consequently better process repeatability. Concerning the laser, we have already demonstrated that by reducing the oxide aperture of the VCSEL to 4 µm we achieved a more circular beam, at the cost of a reduction of the power. An additional approach to improve the beam profile in order to achieve a more Gaussian beam over the VCSELs entire operation range could be given by the integration of the surface relief technique to the device [34]. Indeed, by integrating a (quasi) single-mode VCSEL we expect to achieve a further enhancement in the coupling efficiency for the SMF due to the increase in the overlap integral with the fundamental mode of the fiber. Finally, enhancing the coupling efficiency can be obtained by integrating lenses on both the chip and the fiber termination. In particular, a polymer microlens could be directly fabricated on the VCSEL for the collimation of the beam, hence reducing the divergence angle [35]. Moreover, a metalens with a high numerical aperture could be integrated on the fiber tip to increase the acceptance cone of fiber, therefore better collecting the light [36]. Worth noting, the integration of a flat lens on a SMF is compatible with our bonding process thanks to their subwavelength dimensions. The current results appear of further interest for telecommunication technologies, since standard telecom grade fibers have larger cores than the SMF here investigated. An array of VCSELs can be integrated on the termination of a multimode fiber, provided that it has a core diameter large enough to contain all the oxide apertures of the array. In addition, multiplexing and multisensing capabilities could also be obtained by integrating a VCSEL array on the termination of multicore fibers, ensuring that each laser is coupled into a different core of the fiber.

The new fabrication process can be easily extended to a wide range of optoelectronic devices enabling the development of a new generation of active fiber devices, capable of generating, transmitting, and sensing optical signals in a single optical platform. Such devices could find applications where the technology is currently limited by the presence of benchtop and bulky instrumentation. Examples include assistive and wearable technologies, as well as those concerning ultra-compact point-of-care (POC) detection units. In fact, in the last decades, many multifunctional wearable optical fiber sensors have been developed, which, when mounted onto our clothes or body, continuously monitor and record physiological signals, analyze our body fluids, and capture the sensation of touch for prosthetics, just to name a few [37], [38]. Although the technology for which they interact and communicate with the surrounding environment for health monitoring is well-established, the bulkiness and heaviness of the interrogation equipment connected to the sensors do not allow the technology to be applied in a real-world scenario. A similar argument can be made for other relevant strategic sectors (IoT, personalized medicine) where lightweight and miniaturization of optical components is an important design requirement for the application context [39]. For example, in clinical applications requiring frequent and massive population screening, the use of compact and effective photonic POC units is vital [40]. For fiber optics and its functionality to make a tangible contribution to POC units, it is necessary to avoid the use of cumbersome instrumentation and employ compact light sources and detectors fully combined with optical fiber biosensors. In addition, Wi-Fi modules could be integrated directly into optical fiber ensuring ultra-fast optical communications in IoT systems. Our results may bring advancement also in quantum technology where a mechanically-stable, compact coupling between a light source and a single-mode fiber will sensibly reduce the footprint of the quantum light sources [41]. The current approach would not only allow for an efficient fiber-coupling, but also would ensure the possibility of electrical connecting the sample. The latter is of key importance since the use of diode structures in combination with nanostructures, as semiconductor quantum dots, showed to be key for achieving state-of-the-art emission performances [42], [43].

Overall, the optoelectronics on fiber technology, coupled with advanced fiber sensing could enable advanced lightweight and compact devices capable of sensing and transmitting sensory data under challenging monitoring conditions.

## 5 Methods and Materials

### 5.1 Fiber tip preparation

The optical fibers used for this application are respectively a SMF having a core diameter of 4 µm and a NA of 0.1 (model: SMJ-2.5S2.5S-633-4/125-1-1 from OZ Optics) and a MMF with a core diameter of 105 µm and a NA of 0.22 (model: QMMJ-2.5S2.5S-IRVIS-105/125-1-0.5 from OZ Optics), both with a step-index profile and a ceramic ferrule-top on each termination. To prepare the fibers for the integration of the VCSELS, we modified their terminations with three main steps. First of all, an electron beam evaporation (Kenosistec CL400C) process was used to deposit a gold layer of 150 nm together with an adhesion layer of 10 nm of titanium on both the facet and the lateral side of one termination of the optical fibers. The fibers were mounted on a customized holder in order to have a 60° angle as compared to the evaporation direction to deposit the materials on the tip and on one side of the fiber. The deposition rates were 0.1 and 0.5 nm s^−1^ for the Ti and Au films, respectively. After the deposition, a UV laser micromachining process was used to open a circular hole in the correspondence of the core of the optical fibers. Finally, the thickness of the gold layer was increased to about 1 µm with a galvanization process using a sulphite-based gold bath with a neutral pH-Value, to ensure a more stable bonding of the chip.

### 5.2 VCSEL fabrication

The VCSEL structure was grown by metal-organic vapour-phase epitaxy in an Aixtron 3 × 2 inch close coupled showerhead reactor on a n-doped GaAs wafer with a misorientation of 6° towards the crystalline [111] A direction. The structure contains a n-doped Al_0.50_GaAs:Si/ Al_0.95_GaAs:Si bottom distributed Bragg reflector (DBR) optimized for a high reflectivity in the red spectral range, an active region consisting of GaInP QWs in Al_0.33_GaInP/Al_0.55_GaInP barriers and a p-doped Al_0.50_GaAs:Zn/ Al_0.95_GaAs:Zn top DBR. There, the top DBR inhibits an oxidation layer with high aluminium content close to the active region. Optical lithography with a Karl Suss mjb-3 mask aligner was carried out to define circular masks which were subsequently etched via inductively-coupled plasma etching in order to create the VCSEL mesas. Subsequently, wet-thermal oxidation was used to form the oxide aperture for current confinement. A photosensitive polymer (Cyclotene 4022-35) was used for the isolation layer and the bottom and top metallizations were evaporated by e-beam deposition (for the top p-contact with a prior lithography step.) In the current device layout, each chip has dimensions of about 500 µm × 500 µm, therefore much smaller than the fiber ferrule and contains a single VCSEL located in the center of a large quadratic p-contact pad. Finally, the VCSELs were cleaved into single chips and cleaned in acetone and isopropanol to remove residuals from the cleaving process.

### 5.3 Characterization setup

For free space precharacterization, the VCSEL chip is placed on a temperature controlled copper heat sink which also serves as the n-side electrode. A contact needle probe, controlled by a Karl Suss micromanipulator, is used to contact the top metal pad. For current injection an ILX Lightwave LDP-3811 laser diode driver is used. A biconvex lens with a focal length of 60 mm is applied to collimate the laser emission before it is focused on the detection device. For optical power measurements, a Newport 1830C optical power meter was used. To resolve the spectral behaviour of the VCSEL devices an Ocean Optics HR4000 spectrometer with an optical resolution of 0.75 nm was utilized. For beam profile measurements an additional telescope arrangement of two lenses (f = 150 mm and f = 25.4 mm) in combination with a coherent LaserCam HR II profiling setup was used. After integration on the fiber ferrule, device characterization was carried out by placing the fiber inside a fiber port directly in front of the optical power meter and profiling system, where in the case of spectral measurements a fiber-coupled entrance of the spectrometer was available.

## Supplementary Material

Supplementary Material Details
