# Supplementary material for: Monolithic integration of one VCSEL on a single mode fiber
Source: Nanophotonics. 2025 Jun 23;14(14):2431–42. doi: 10.1515/nanoph-2025-0047 (PMC12273538; doi:10.1515/nanoph-2025-0047)
Supplement: Supplementary file 1 — Supplementary Material Details [file j_nanoph-2025-0047_suppl_001.docx]

**Supporting Information**


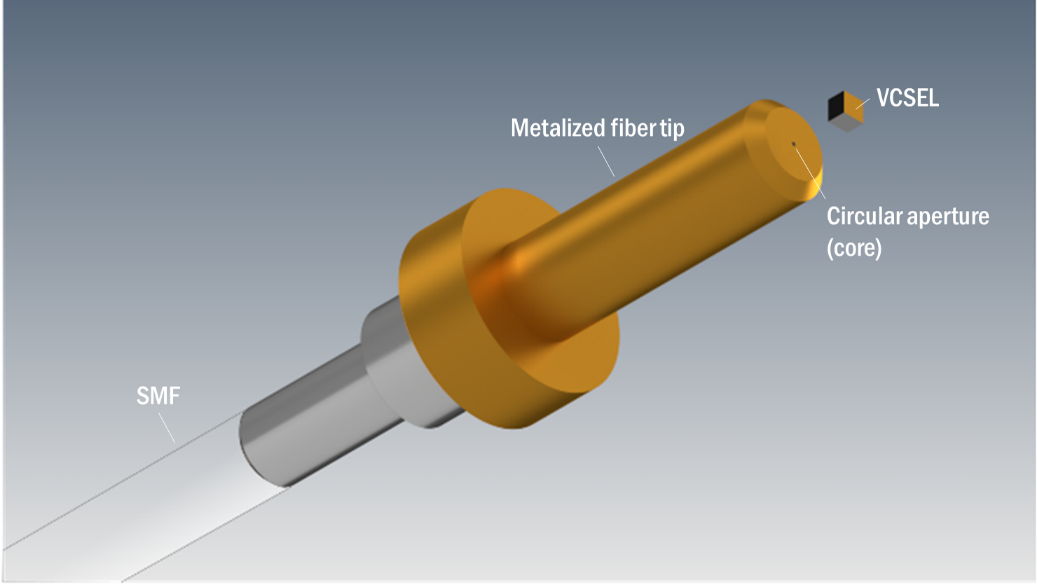


*Figure S1: Illustration of the proposed fiber-integrated VCSEL.*

***Fiber Theory***

In the case of SMF, the fundamental mode propagating in the SMF is simply given by:

$E_{f}\left( x,y,z \right)=\frac{\sqrt{2/\pi}}{\omega_{f}} e^{-\frac{x^{2}+y^{2}}{\omega_{f}^{2}}} e^{-jkz}$ (SI1)

where $\omega_{f}$is the mode-field radius of the fiber.

For the MMF, starting from the core diameter, NA, and step-index profile of the selected optical fiber, the refractive index of core and cladding and the V number (Equation SI2) can be estimated. The higher the V number of the fiber, the more guided modes exist.

$V=\frac{2\pi}{\lambda}r_{core} NA=\frac{2\pi}{\lambda}r_{core} \sqrt{n_{core}^{2}k^{2}-\beta^{2}}$ (SI2)

In particular, the complex electric field profile $E(r,\varphi)$ in cylindrical coordinates (Equation 3), where $k=2\pi n/\lambda$ is the wavenumber resulting from the local refractive index n and the vacuum wavelength $\lambda$, for the specific case of step-index fibers (where the refractive index is constant within the [fiber core](https://www.rp-photonics.com/fiber_core.html)), has analytical solutions for the core and [cladding](https://www.rp-photonics.com/fiber_cladding.html) part that can be expressed as a Bessel function (Equation SI4) and a modified Bessel function (Equation SI5) respectively.

$\frac{\partial^{2}E}{\partial r^{2}}+\frac{1}{r}\frac{\partial E}{\partial r}+\frac{1}{r^{2}}\frac{\partial^{2}E}{\partial\varphi^{2}}+\frac{\partial^{2}E}{\partial z^{2}}+k^{2}E=0$ (SI3)

$u=r_{core}\sqrt{n_{core}^{2}k^{2}-\beta^{2}}$ (SI4)

$w=r_{core}\sqrt{\beta^{2}-n_{clad}^{2}k^{2}}$ (SI5)

***Technical details: characterization of the devices***

***Custom electrical plug***


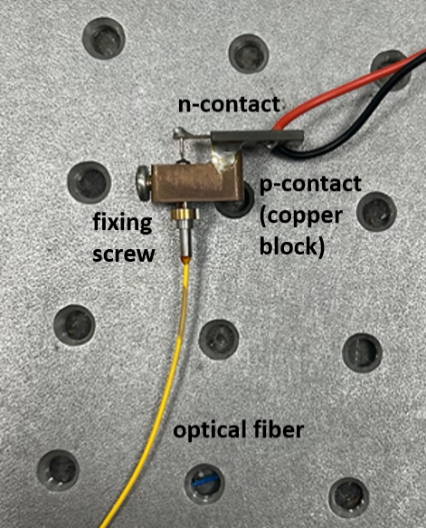


*Figure S2: Image of the fiber-integrated device plugged in the custom electrical PCB used for the characterization.*

The “customized electrical PCB” realized to control the current injection in the fiber integrated device presents a copper block to contact the gold coated termination of the fiber (lateral side of the ferrule) and a needle to contact the backside of the VCSEL.

***Optical Spectra***

*Figure S3: Optical spectra of the 4 selected VCSELs measured in free space with a temperature of the heatsink of T=15°C, at the current of thermal rollover.*

***Output power over injected current***

Figure S4: Measured P-I curves before and after (black curves) the integration on the fibers of (a) VCSEL 1, (b) VCSEL 2, (c) VCSEL3, (d) VCSEL 4.

***Estimated coupling efficiency***

**

Figure S5: Coupling efficiency as a function of the current, for the (a) VCSEL 1, (b) VCSEL 2, (c) VCSEL 3, (d) VCSEL 4.

Figure S6: Coupling efficiency calculated with the power levels method for the (a) VCSEL 1, (b) VCSEL 2, (c) VCSEL 3, (d) VCSEL 4. The P-I experimental curves were interpolated to obtain the blue/green (free space) and black (on fiber) curves.

Figure S5 shows the experimental coupling efficiency estimated for each current value. The coupling efficiency values are affected by changes in the threshold current resulting from integration, especially for low powers (or currents).

Figure S6 shows the experimental coupling efficiency estimated for each value of emitted power normalized with respect to that relative to rollover.

Figure S7: Coupling efficiency as a function of emitted power levels normalized to the values.

Figure S7 shows the coupling efficiency trends for all fibers from which the values at rollover were subtracted to make the comparison more straightforward. The percentages shown in the x-axis represent the decreases in emitted powers expressed as percentages from the values at rollover (corresponding to 100%). Interestingly, the trends obtained for the four fibers are very similar to each other; as one moves away from rollover, the efficiency tends to increase exponentially (a slightly stronger trend is seen for SMFs). This is because for low injection currents the laser beam tends to be more Gaussian leading to an increase in coupling efficiency in line with the numerical model.

***Selecting beam waist***

For each VCSEL, the theoretical waist was estimated by taking into account the experimental beam profiles and the oxide aperture dimensions of the four VCSELs. For this reason, the waists estimated for VCSELs integrated on MMFs are bigger than the ones on SMFs, and this difference is reflected in the achieved coupling efficiencies. In particular, we selected a waist of 2.0 µm and 1.7 µm for VCSEL 1 and 2 respectively. Since the waist chosen for the first device is closer to the threshold value predicted to achieve the maximum coupling efficiency for the MMF, its coupling efficiency is higher than the one of VCSEL 2. A difference in the simulated coupling efficiency can also be observed for the other two devices on SMFs, with a lower value obtained for VCSEL 4 in comparison to VCSEL 3. Indeed, even if the waists selected for both devices are below the optimal value of 2.5 µm calculated from the parametric study, explaining why their theoretical coupling efficiencies don’t reach higher values, the waist of 1.5 µm estimated for VCSEL 3 is 0.5 µm bigger than the one chosen for VCSEL 4.

Table S1:

|  | VCSEL 1 | VCSEL 2 | VCSEL 3 | VCSEL 4 |
| --- | --- | --- | --- | --- |
| Oxide apertures [µm] | 4.75 x 5.99 | 5.05 x 6.34 | 3.5 x 2.9 | 3.1 x 2.7 |
| Measured waist (low current) [µm] | 2.0 | 1.6 | 1.7 | 1.3 |
| Estimated waist (rollover) [µm] | 1.9 | 1.5 | 1.6 | 1.2 |

***Selecting modes percentages***

**
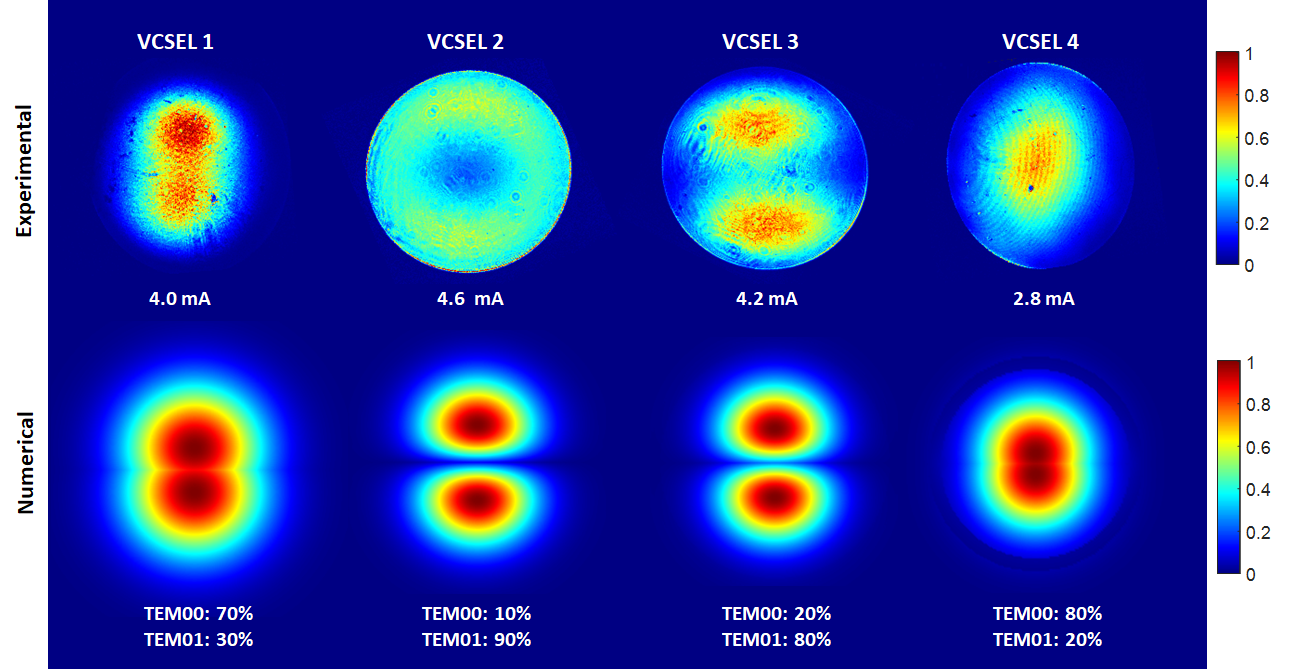
**

Figure S8: Experimental beam profile measured in free space at 15°C of the four selected VCSELs (top). Numerical beam profiles obtained by different percentages combination of ${TEM}_{00}$, ${TEM}_{01}$, and ${TEM}_{10}$ modes (bottom).

The beam profiles measured at (or near) thermal rollover were analyzed and different combinations of the HG modes were tested in order to achieve a similar beam profile with the numerical simulations. Since $\mathrm{TEM}_{01}$ and $\mathrm{TEM}_{10}$ modes give the same contribution and the percentage associated with the $\mathrm{TEM}_{11}$ mode is negligible, in our calculations, we considered a linear combination of only $\mathrm{TEM}_{00}$ and $\mathrm{TEM}_{01}$, changing the percentage of the latter between 0% (i.e. 100% $\mathrm{TEM}_{00}$, that is full Gaussian mode) to 100%. The hypothesized percentages (reported in the above figure) were used as a starting point to compare the estimated coupling efficiency at thermal rollover, of the four devices, with the numerical simulations, as shown in Figure 7 (a). Since the experimental observations do not allow us to derive the precise value of these percentages, they were varied to the extent of 10 percent and the results are shown as error bars in the graph below.

******

Figure S9: Theoretical coupling efficiencies for the four devices varying percentages combination of the modes.

***Speckle Modeling***

Figure S8 shows the laser beam in free space (top) and at the other extremity of the fiber post-integration (below). While for the SMFs the typical Gaussian behavior is shown, the output of the MMFs exhibits the classical speckle pattern due to the interference arising from the thousands of modes excited into the fiber.

Figure S10: Beam profile after the integration on the optical fiber and Speckle modeling.

We simulated the beam propagation through both the MMF and the SMF (Figure S9). The speckle at the end of the fiber was modeled as a randomization of the modes coupled and propagating into the fiber, demonstrating the appearance of the speckle for a MMF and its absence for a SMF.

Figure S11: Simulated output beam at the end of a MMF (left) and a SMF (right).
